# Patient outcomes after electrical injury – a retrospective study

**DOI:** 10.1186/s13049-021-00920-3

**Published:** 2021-08-06

**Authors:** Jamal Ahmed, Charlotte Stenkula, Sherwan Omar, Josef Ghanima, Fredrik Førsund Bremtun, Jonas Bergan, Nezar Raouf, Waleed Ghanima

**Affiliations:** 1 Department of Emergency Medicine, Østfold Hospital Trust, Gralum, Norway; 2 Department of Research Østfold Hospital Trust, Gralum, Norway; 3 Department of Hematology, Oslo University Hospital and Institute of Clinical Medicine, University of Oslo, Oslo, Norway

**Keywords:** Electrical injury, Arrhythmia, High voltage

## Abstract

**Introduction:**

People exposed to electrical injuries are often admitted to hospital for observation. Current evidence suggests that patients who have a normal ECG on admission after a low-voltage injury, with no loss of consciousness or initial cardiac arrest may be discharged home after a short observation time. Currently, there are no established standards for the duration of monitoring after electric shock, but 24 h of observation is the most commonly adopted approach. We carried out a retrospective study of patients admitted after electrical injuries to determine the in-hospital outcomes and 30-day mortality in these patients.

**Methods:**

We performed a chart review of all patients with electrical injuries admitted to Østfold Hospital, Norway between the years 2001 and 2019, to determine in-hospital and 30-day mortality and the frequency of various cardiac and non-cardiac complications.

**Results:**

Mean age of 465 included patients (88% males) was 31 years. Of all injuries, 329 (71%) were work-related, 17 (3.7%) involved loss of consciousness. Furthermore, 29/437 (6.6%) were high voltage (> 1000 V), and 243/401 (60.6%) were transthoracic injuries.

369 (79.4%) were discharged same day. None of the admitted patients died in hospital nor did any die within 30 days of admission, yielding a 30-day mortality of 0% (95% CI 0–0.8). At admission troponin was elevated in three (0.6%) patients, creatinine kinase (CK) in 30 (6.5%) and creatinine in six (1.3%). Electrocardiogram (ECG) abnormalities were described in 85 (18%) patients. No serious arrhythmias were detected.

When comparing high- vs low-voltage or transthoracic vs other injuries, there were no significant differences between most of the outcomes, except for more ECG abnormalities in the transthoracic group, whereas more patients had elevated CK, and fewer discharged the same day in high-voltage injuries.

**Conclusion:**

No in-hospital nor 30-day mortality or serious arrhythmias were encountered in those who were assessed, regardless of the type of injury. Troponin and creatinine were rarely elevated. It seems that conscious patients admitted with a normal ECG following a low-voltage injury may safely be discharged home after a quick clinical assessment including ECG.

## Background

Electrical injury occurs when a person becomes part of an electrical circuit. The damage caused by electrical injuries can range from minor skin burns to life-threatening damage to vital organs. Exposure to electrical injuries can be caused by lightning strikes, electricity at home or at the workplace [1–3]. According to the Occupational Safety and Health Administration, the statistics indicate that electrical injuries are the third leading cause of occupation-related deaths in the United States [2]. People exposed to electrical injuries are often admitted to hospital for observation. Current evidence suggests that patients who have a normal ECG on admission after a low-voltage injury, short contact with the electrical source and with no loss of consciousness or initial cardiac arrest may be discharged home after a quick clinical assessment [1, 4–8]. A normal ECG seems to predict absence of late arrhythmias [8–10]. However, there are no established standards for the duration of monitoring after an electric shock, but 24 h of observation is the most commonly adopted approach [1]. There are several factors defining the severity of damage after an electrocution. The most important factors are the amount of electrical current flow, most commonly defined as voltage above or below 1000 V [1, 3]. Electrical current > 1000 V greatly increases the mortality rate [3]. Another important factor is the pathway of which the current travels. Transthoracic pathways have the highest mortality rate due to the potential for increased spinal cord, and myocardial damage [11]. Other factors include the type of tissue the electrical current travels thorough where wet or moist skin increases the conduction and causes more severe injuries, the duration of contact with the current and lastly the type of current, where alternating current is believed to be more dangerous because it can cause tetanic muscle contractions.

Due to the paucity of evidence regarding the optimal management of patients exposed to electrical injuries, we carried out a retrospective study of patients admitted due to electrocution. The aims of the study were to determine patients’ characteristics and outcomes of electrical injuries.

## Methods

### Study design

This is a retrospective observation study with a chart review of patients admitted because of electrocution. The aim of the study was to determine patients’ characteristics and outcome of electrocution.

### Setting

All individuals referred to the emergency departments and/or admitted at Østfold hospital with electrocution, between 2001 and 2019, were identified and included in the study. Østfold hospital is a primary referral center for Østfold County. The hospital has a catchment area of 317,000 individuals. In 2020 a total of 45,498 patients were admitted to Østfold hospital and 420,322 outpatient consultations were performed.

### Participants

To identify the study individuals we searched the hospital administrative system using ICD-10 diagnostic codes. All individuals > 18 year with the following ICD-10 codes: T75.0 unspecified effects of lightning and T75.4 electrocution were identified and included. Our only exclusion criterion was age below 18 years.

Electrical injuries were classified and grouped into transthoracic (defined as current traveling from one upper extremity to the other or from one upper extremity to a lower extremity or travelling directly through the thoracic region) and non-transthoracic injuries as well as high voltage (> 1000 V) and low voltage injuries (< 1000 V). In the group of identified patients, following outcomes were investigated: death within hospital stay and within 30 days after injury; same day discharge (discharged before midnight same day) or admission longer than one day; loss of consciousness; ECG changes including arrhythmias, or any ECG change not present previously; biochemical parameters or any other injury including skeletal damage or burns.

### Variables

Biochemical parameters were extracted from laboratory databases. Quantitative real-time cardiac Troponin-T or Troponin-I acquired at admission were recorded. Since different assays were used during the study periods, this outcome was dichotomized into positive or negative. Other blood tests included creatinine kinase, CK-MB, creatinine, calcium and potassium. All results were dichotomized into positive or negative.

### Data sources

The chart review was conducted by 4 investigators (JA, SO, JG, FFB). Following the identification of study individuals, the reviewers reviewed patients’ electronic records including laboratory and radiological investigations to capture and complete a standardized case report form that was developed for this study. The work was divided equally between the reviewers and the charts were reviewed only once. Data were then plotted into electronic database to perform statistical analysis. The ECG findings were reviewed and interpreted by the investigators, and then checked against the initial evaluation. ECG with abnormal findings were reviewed in addition by a cardiologist (NR).

### Statistics

Categorical variables were expressed as numbers and proportions, whereas continuous variables were presented as mean and standard deviation (SD). Statistical significance between groups were tested either using two-sided Fisher’s exact test (categorical variables) or Mann Whitney *U* test for skewed continuous variables, respectively. *P*-value < 0.05 was considered statistically significant.

Since the study was a quality control study, the regional ethics committee provided exemption from obtainment of patients consents.

## Results

We identified 465 patients admitted to the emergency department or other departments during the study period. Mean age was 31 years (SD ± 11); of these 410 (88%) were men. Seventeen (3.7%) patients experienced loss of consciousness, 26 (7.7%) experienced tetany or cramps, 38 (8.2%) reported chest pain and 56 (12%) had skin burns. No skeletal injuries were observed.

These injuries were classified as work-related in 329 (71%), non-work-related in 107 (23%) whereas the cause was unknown in 29 (6%). The voltage was specified in 437 patients; of these 29 (6.6%) were classified as high-voltage and the remaining were low-voltage. Injuries were classified as transthoracic in 243 of 401 (60.5%) patients.

Three hundred and sixty-nine (79%) patients were discharged from hospital same day as admitted; 90 (19%) patients were discharged the next day, while the remaining six patients (1.3%) stayed for up to 8 days. None of the admitted patients died in hospital, nor did any die within 30-days of admission, yielding a 30-day mortality of 0% (95% CI 0–0.8).

At admission troponin was elevated in three (0.6%) patients, CK in 30 (6.5%) CK-MB in one (0.2%), and a new creatinine rise was seen in six (1.3%) patients. Of note, all the patients with elevated creatinine were at the age of 55 years or older. Apart from one patient who had a known chronic renal failure, the other five had only marginally elevated creatinine [highest creatinine value was 122 umol/l (upper normal limit 105 umol/l]. CK was elevated in two of the five patients. Furthermore, serum potassium was elevated in one (0.2%) and serum calcium in two (0.4%) patients.

ECG abnormalities were observed in 85 (18%) patients. The most common ECG abnormality was ST-T changes (11%); however, all of these were minor and were classified as clinically irrelevant. In all cases, the ECG abnormalities were asymptomatic and did not require any intervention. Other changes included non-significant ST-elevations, ventricular or supraventricular extra systole, previously diagnosed normal frequency atrial fibrillation, sinus tachycardia or sinus bradycardia (Tables 3 and 4).

Patients who had a transthoracic electrical injury were more frequently observed with abnormal ECG (22% vs 13%), had more often chest pains (12% vs 0.6%) and cramps (10% vs 2%) compared to patients with no transthoracic injury (Table 1). There was no statistical difference between the rates of same day discharge in patients with transthoracic injury [(195 of 243 (80%)] compared to those with no transthoracic injury [(128 of 158 (81%); *p* = 0.8].

**Table 1:** Outcomes based on transthoracic vs. no transthoracic injury

	Transthoracic injury *n* = 243	No transthoracic injury *n* = 158	*p*-value
Age, years; mean (SD)	30 (11)	31 (12)	0.95
Male; *n* (%)	222 (91)	131 (83)	0.02*
Work related; *n* (%)	182 (75)	109 (69)	0.14
**Clinical manifestations**			
Loss of consciousness; *n* (%)	10 (4.1)	2 (1.3)	0.13
Tetany/convulsion/cramps; *n* (%)	24 (10)	3 (2.0)	0.001**
Chest pains; *n* (%)	28 (12)	1 (0.6)	0.001**
Skin burns; *n* (%)	25 (10)	19 (12)	0.87
ECG abnormal; *n* (%)	54 (22)	20 (13)	0.02*
**Outcomes**			
CK elevated; *n* (%)	14 (5.8)	9 (5.7)	1
CK-MB elevated; *n* (%)	1 (0.4)	0 (0)	1
Troponin I elevated; *n* (%)	0 (0)	3 (2.0)	0.05
Potassium elevated; *n* (%)	1 (0.4)	0 (0)	1
Calcium elevated; *n* (%)	2 (0.8)	0 (0)	0.52
Creatinine elevated; *n* (%)	3 (1.2)	1 (0.6)	1

In those exposed to high-voltage electrical shock, elevated levels of CK were significantly higher compared to the patients exposed to lower voltage electrocution (Table 2). There were significantly lower rates of same day discharge and longer admission in patients exposed to high-voltage electrical shock [(15 of 29 (50%)] compared to low-voltage [(334 of 407 (82%); *p* = 0.0001].

**Table 2:** Outcomes based on high-voltage vs low-voltage injury

	High-voltage *n* = 29	Low-voltage *n* = 408	*p-*value
Age, years; mean (SD)	34 (12)	31 (11)	0.14
Male; *n* (%)	26 (90)	359 (88)	1
Work related; *n* (%)	17 (59)	297 (73)	0.34
**Clinical manifestations**			
Loss of consciousness; *n* (%)	3 (10)	13 (3.2)	0.08
Tetany/convulsion/cramps; *n* (%)	3 (10)	29 (7.1)	0.44
Chest pains; *n* (%)	3 (10)	32 (7.8)	0.44
Skin burns; *n* (%)	7 (24)	43 (11)	0.06
ECG abnormal; *n* (%)	8 (28)	68 (17)	0.13
**Outcomes**			
CK elevated; *n* (%)	7 (24)	21 (5.1)	0.002**
CK-MB elevated; *n* (%)	0 (0)	1 (0.2)	–
Troponin I elevated; *n* (%)	1 (3.4)	2 (0.5)	0.19
Potassium elevated; *n* (%)	0 (0)	1 (0.2)	1
Calcium elevated; *n* (%)	0 (0)	2 (0.5)	–
Creatinine elevated; *n* (%)	1 (3.4)	5 (1.2)	0.37

**Table 3:** ECG changes seen in relation to transthoracic vs. no transthoracic injury

	Transthoracic injury	No transthoracic injury	*p*-value
Arrhythmia; n (%)	9 (3.7)	3 (1.9)	0.74
Supraventricular disturbance; n (%)	10 (4.1)	2 (1.3)	0.31
Ventricular disturbance; n (%)	11 (4.5)	6 (3.8)	0.76
ST-T changes; n (%)	35 (14)	11 (7.0)	0.20

**Table 4:** ECG changes seen in relation to high-voltage vs. low-voltage injury

	High voltage	Low-voltage	*p*-value
Arrhythmia; n (%)	2 (6.9)	9 (2.2)	0.62
Supraventricular disturbance; n (%)	3 (10)	12 (2.9)	0.38
Ventricular disturbance; n (%)	2 (6.9)	15 (3.7)	1
ST-T changes; n (%)	3 (10)	43 (11)	0.15

## Discussion

Our data reveals no in-hospital mortality or 30-day mortality or serious arrhythmias encountered in those admitted to the emergency department of our hospital. These findings are similar to other studies that have shown generally favorable outcomes from these types of injuries [4–6, 12, 13]. In 2019, Pilecky et al. published a single center study on the risk of developing cardiac arrhythmias after electrical accidents. This study included 480 patients with a 0% 30-day mortality rate. Sinus bradycardia and sinus tachycardia were the most frequent ECG abnormalities. All of them were asymptomatic and none required any intervention [10]. A Danish nationwide cohort study examined whether electrical shock patients had an increased risk of developing cardiac disease, arrhythmias or death compared with the general Danish population [14]. This study included 11,462 patients following an electrical injury between the period of 1994 and 2011. The results had a 5-year cumulative mortality rate of 0.47% showing no difference in 5-year survival compared with matched controls. The study also concluded that late cardiac arrhythmias following an electrical injury were rare.

As in previously reported studies, we can also conclude that the majority of patients exposed to electrical injury are of male gender, younger age, and had work-related injuries [6, 10, 15]. We also observed that 93% of our patients were exposed to low-voltage injuries. Our results show that, when comparing high- vs low-voltage or transthoracic vs no transthoracic injuries, there were no significant differences between most of the studied outcomes. The only significant differences we found, were a greater number of patients with ECG abnormalities and tetany/cramps in the transthoracic group, and more patients had elevated CK in high-voltage injuries compared to the low-voltage, as described in Tables 1 and 2 [16]. In addition, we found a significantly lower rate of same day discharge in patients exposed to high- voltage injuries. Of note, troponins and creatinine levels were rarely elevated regardless of the type of injury. Of those with elevated creatinine, only one had known chronic renal failure. However, all the patients with elevated creatinine were above the age of 55, which may possibly be explained by the presence of some previous renal impairment rather than the electrical injury itself. However, since the vast majority of patients had low-voltage injuries, our results cannot be extrapolated to high-voltage injuries.

Looking at arrhythmias, previous publications claim that clinically relevant arrhythmias are rare after an electrical injury [6–10, 14]. Arrhythmias caused by electrical shock usually occur immediately after an electrical injury, and only a few cases of late onset arrhythmias have been reported [5, 7, 9, 10, 13]. In our study, 18% of patients had ECG changes. However, all of these changes were minor, and no treatment was required. Unfortunately, very few of our patients had previous ECGs in our hospital electronic patients’ record systems; thus, it was impossible to conclude whether the ECG changes are of new onset or were present prior to the injury. One may also suspect that abnormalities like sinus tachycardia may be explained by a psychological response to the injury, like anxiety or pain [10]. Although some ECG abnormalities were observed, no serious arrhythmias were encountered, and all changes could be classified into clinically irrelevant ones.

Finally, 12 patients had burns; all of which were minor burns not requiring any surgical intervention.

To our knowledge, this study is one of the largest studies conducted on outcomes after an electrical injury. We used an unselected population of patients that represent electrical injuries admitted to a general emergency department. The main limitations to this study include that it was a retrospective data collection and patients were not observed systematically after the accident. Further, potential delayed complications such as arrhythmias may have occurred without being captured or registered. However, the 30-day mortality was null, which is reassuring and excluded the possibility of serious late complications. Lastly, another limitation involves the variable time between the accident and biochemical analyses that may have influenced our results.

## Conclusion

**Fig. 1:**
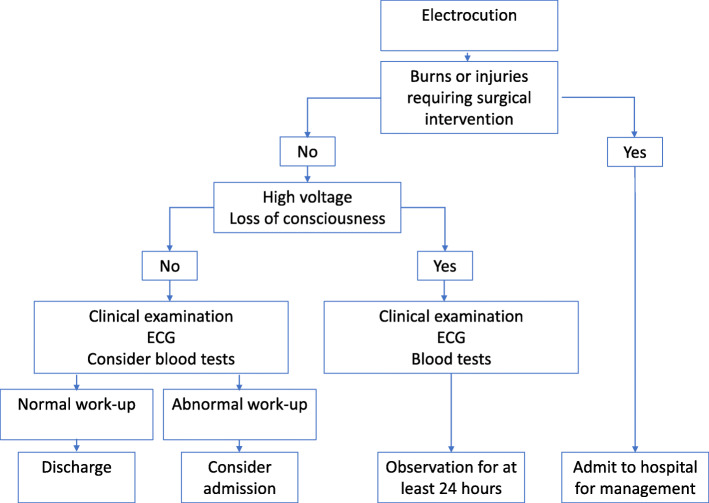
Proposed algorithm for the in-hospital management of patients exposed to electrocution

We conclude that conscious patients admitted with a normal ECG following a low- voltage electrical injury are at a low risk of mortality and may safely be discharged home after a quick clinical assessment. Conversely, high-risk patients including those with ECG-changes, loss of consciousness and/or high-voltage injuries, will still be recommended for a 24-h observation before being discharged home. Based on our findings and current literature we propose an algorithm for the in-hospital management of patients exposed to electrocution (Fig. 1).

## Data Availability

All data used or analyzed in this study are available from the corresponding author upon reasonable request.
